# *Clostridium perfringens* Sialidases: Potential Contributors to Intestinal Pathogenesis and Therapeutic Targets

**DOI:** 10.3390/toxins8110341

**Published:** 2016-11-19

**Authors:** Jihong Li, Francisco A. Uzal, Bruce A. McClane

**Affiliations:** 1 Department of Microbiology and Molecular Genetics, University of Pittsburgh School of Medicine, Room 420, Bridgeside Point II Building, 450 Technology Drive, Pittsburgh, PA 15219, USA; jihongli@pitt.edu; 2 California Animal Health and Food Safety Laboratory, San Bernardino Branch, School of Veterinary Medicine, University of California-Davis, San Bernardino, CA 92408, USA; fauzal@ucdavis.edu

**Keywords:** *Clostridium perfringens*, intestinal infections, gas gangrene, toxins, sialidases, sialidase inhibitors

## Abstract

*Clostridium perfringens* is a major cause of histotoxic and intestinal infections of humans and other animals. This Gram-positive anaerobic bacterium can produce up to three sialidases named NanH, NanI, and NanJ. The role of sialidases in histotoxic infections, such as gas gangrene (clostridial myonecrosis), remains equivocal. However, recent in vitro studies suggest that NanI may contribute to intestinal virulence by upregulating production of some toxins associated with intestinal infection, increasing the binding and activity of some of those toxins, and enhancing adherence of *C. perfringens* to intestinal cells. Possible contributions of NanI to intestinal colonization are further supported by observations that the *C. perfringens* strains causing acute food poisoning in humans often lack the *nanI* gene, while other *C. perfringens* strains causing chronic intestinal infections in humans usually carry a *nanI* gene. Certain sialidase inhibitors have been shown to block NanI activity and reduce *C. perfringens* adherence to cultured enterocyte-like cells, opening the possibility that sialidase inhibitors could be useful therapeutics against *C. perfringens* intestinal infections. These initial in vitro observations should be tested for their in vivo significance using animal models of intestinal infections.

## 1. An Introduction to *Clostridium perfringens*

*Clostridium perfringens* is present throughout the environment, including soil, sewage, feces, foods, and the normal gastrointestinal flora of animals [1,2]. This Gram-positive, anaerobic, spore-forming bacterium is also a feared pathogen of both humans and other animals [2,3]. The most notable *C. perfringens* histotoxic infection is the rapidly-fatal human disease named clostridial myonecrosis (traumatic gas gangrene) [4,5]. This bacterium is also a preeminent cause of common, and sometimes lethal, infections originating in the intestines of humans or livestock [2,6]. Those intestinal infections often involve damage to the small intestine, or to both the small intestine and colon, which results in enteritis or enterocolitis, respectively [1,2]. *C. perfringens* intestinal infections can also progress to enterotoxemia, where a toxin(s) is produced in the intestines and then absorbed to affect extraintestinal organs such as the brain [2,7]. 

The virulence of this bacterium involves its ability to produce a vast toxin armory [2,3,8]. Currently ~20 different *C. perfringens* toxins have been identified, with more likely awaiting discovery [8,9,10,11,12,13,14,15]. Toxin production repertoires vary greatly among different *C. perfringens* strains, permitting classification of these isolates into five types (A–E), based upon an isolate’s production of four typing toxins (alpha, beta, iota, and epsilon toxins) (Table 1) [9,10]. 

*C. perfringens* type designations correlate with disease causation, as shown in Table 2. Two typing toxins, i.e., beta toxin (CPB) and epsilon toxin (ETX), have proven importance in *C. perfringens* intestinal infections of mammalian livestock [3,16,17]. *C. perfringens* produces other toxins that, while not used for typing classification, are nonetheless important for infections originating in the intestines of agriculturally-important animals. The foremost example is necrotic enteritis B (NetB) toxin, which is critical when *C. perfringens* causes avian necrotic enteritis in poultry [12]. 

With respect to human *C. perfringens* infections, type A strains are responsible for causing most histotoxic infections. During gas gangrene, alpha toxin (CPA) plays the major role in virulence. A non-typing toxin named PFO also contributes to this disease [4,5]. 

To date, only type A and C strains of *C. perfringens* have been conclusively linked to human diseases originating in the intestines [1,2,3,18]. Type C strains use their CPB to cause enteritis necroticans (EN), which was first described in post-World War II Germany, where it was referred to as darmbrand [18,19,20]. In the 1960s–1970s, EN, known locally as pigbel, was a major cause of death of children in the Papua New Guinea (PNG) Highlands [20,21]. Pigbel develops in children with reduced trypsin levels due to predisposing conditions, including malnutrition, a diet rich in sweet potato (which contains a trypsin inhibitor), and/or intestinal infections with pathogens producing a trypsin inhibitor [20,21]. Their low intestinal trypsin levels render these children susceptible to infection by type C strains because normal trypsin levels would otherwise easily inactivate CPB when it is produced in the intestines. Consequently, children suffering from pigbel develop CPB-induced necrotic enteritis or enterotoxemia and often die rapidly. The only treatment for pigbel is resection of the bowel; however, this surgical intervention is only effective if performed early after the onset of infection [20,21]. A vaccine introduced in the 1980s dropped the incidence of pigbel dramatically in PNG. Unfortunately, pigbel vaccination has since decreased and this illness may now be reappearing. 

Although not used for typing classification, CPE is the toxin responsible for causing the gastrointestinal symptoms of *C. perfringens* type A food poisoning (FP) [1,22]. This FP is currently the 2nd most common bacterial foodborne disease in the USA, where one million cases occur annually and economic losses approach $500 million/year [1,23]. In people with fecal impaction or severe constipation due to side-effects from medications used to treat other pre-existing conditions, *C. perfringens* type A FP can be much more severe and is often fatal [1,24]. Studies with animal models suggest this may be due to absorption of CPE from the intestines, resulting in an enterotoxemia that involves the liver and kidneys [24]. CPE-producing type A strains also cause about 5%–15% of all cases of nonfoodborne human GI diseases, most notably antibiotic-associated diarrhea (AAD) [1,25]. 

As discussed later, CPE-associated AAD cases are more severe and longer lasting than typical cases of *C. perfringens* type A FP, which usually self-resolve within 24 h [1,25]. While the *cpe* gene can be either chromosomal or plasmid-borne in type A strains, most (~70%) type A FP strains carry a chromosomal *cpe* gene [1]. In contrast, nearly 100% of type A AAD strains carry a plasmid *cpe* gene [1]. There are also many other genetic differences between these two groups of type A *cpe*-positive groups. For example, type A strains carrying a chromosomal *cpe* gene produce a unique small acid soluble protein (SASP4) variant that provides their spores with greater resistance to heat, cold, and chemical treatment and probably facilitates survival of these bacteria in the food environment [1,26,27]. Other differences between type A chromosomal *cpe* strains and type A plasmid *cpe* strains, particularly with regard to sialidase gene carriage, will be discussed later. 

In addition to its essential role during intestinal infections by type A *cpe*-positive strains, CPE may also contribute to some cases of human EN caused by type C strains [18]. However, CPB is clearly of critical importance for the pathogenesis of this disease [16]. 

## 2. *C. perfringens* Sialidases

Sialic acids are a carbohydrate family containing about 40 different nine-carbon relatives of neuraminic acid [28,29]. Under physiological conditions, sialic acids are negatively charged [29]. N-acetylneuraminic acid (Neu5Ac), whose amino group is acetylated, is the most widespread sialic acid [29,30]. Sialic acids are important components of the serum and mucus and represent the terminal sugar residue of many glycan chains on host cell surfaces, where they are involved in cell- cell recognition. Sialic acids can also stabilize enzymes or cell membrane proteins. Finally, due to their negative charge, sialic acids can mediate binding and transport of positively-charged molecules [28,29,30]. 

Sialidases, also referred to as neuraminidases (E.C.3.2.1.18), are key enzymes that hydrolyze the α-linkage of terminal sialic acids on various sialoglycoconjugates to generate free sialic acid [28,29,30,31]. Sialidases are made by certain viruses, microorganisms, and vertebrate animals, but not by plants. Included amongst the sialidase-producing viruses and bacteria are several prominent pathogens, e.g., influenza virus, *Vibrio cholerae*, *Streptococcus pneumoniae*, and *C. perfringens*. Sialidases can have a nutritional function for normal flora or pathogenic bacteria [28,29,31]. In addition, they often function directly as virulence factors during bacterial pathogenesis [30,32], as will be discussed later for *C. perfringens*. 

**Figure 1:**
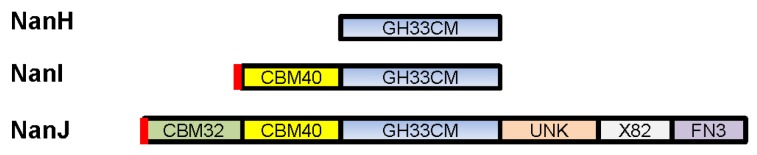
Modular organization of the *C. perfringens* sialidases. CBM32 is a carbohydrate binding module (CBM) belonging to the family 32 CBM; CBM40 is a module having an amino acid sequence identity with a family 40 CBM; GH33CM is a family 33 CBM; UNK is a module having unknown function; X82 is a family 82 “X module” of unknown function; FN3 is a module sharing distant identity with fibronectin type III domains. The red small boxes are secretion signals. Modified with permission from [35]. Copyright 2007 American Chemical Society.

*C. perfringens* produces three different sialidases, which are named NanH, NanI, and NanJ [33]. NanH (43 kDa) lacks a secretion signal peptide and thus has a cytoplasmic location in log-phase cultures [33,34]. In contrast, NanI (77 kDa) and NanJ (129 kDa) are secreted exosialidases ([33], Figure 1). The catalytic modules of all three sialidases show conserved amino acid sequence identity and belong to the family 33 carbohydrate binding module (CBMs, Figure 1) [35]. Compared to NanH, which only consists of a catalytic domain, NanI and NanJ also possess additional accessory carbohydrate-binding modules [35]. It is thought that these carbohydrate-binding domains increase the binding affinity between NanI and NanJ and their polyvalent substrates. NanJ has a complex multimodular structure comprised of a central catalytic module and five accessory modules [35]. The two N-terminal modules show amino acid sequence identity with family 32 and family 40 CBMs. NanI has a simpler structure consisting of a catalytic module and an N-terminal family 40 CBM [35]. 

Most *C. perfringens* strains produce all three sialidases (further discussion below). However, as discussed in more detail later, some *C. perfringens* strains produce only one or two of the three sialidases. For strains producing all three sialidases, NanI is usually responsible for ~70% of total exosialidase activity [33,34,36,37]. 

To characterize the properties of the three *C. perfringens* sialidases, a recent study constructed a series of isogenic mutants, where two of the three sialidase genes present in *C. perfringens* type D strain CN3718 were inactivated [33]. This strategy created mutants that were each expressing, at their native levels, only NanJ, NanI, or NanH in a background free of contamination from the other two sialidases to allow a precise characterization of the enzymatic properties of each sialidase. NanI was found to be more heat-tolerant compared to NanJ or NanH, both of which exhibited greatly reduced sialidase activity at temperatures above 43 °C [33]. In this experimental system, all three sialidases worked best at low pH conditions (pH ~ 5). The enzyme activity of each sialidase was shown to vary in sensitivity to various metal ions [33]. Furthermore, unlike the sialidases from *Streptomyces* spp., *C. perfringens* sialidases were found to be sensitive to p-chloromercuribenzoate, which reacts with thiol groups in proteins [33]. Finally, the three *C. perfringens* sialidases showed different substrate preferences. NanI exhibited preferential activity in the order of α-2,3 > α-2,6 > α-2,8 sialic acid linkages and was responsible for most of the activity in CN3718 supernatants that was directed against those sialic acid linkages. NanJ showed a preference for α-2,6 > α-2,8 > α-2,3 sialic acid linkages. Finally, NanH activity was strongest for α-2,8 > α-2,3 > α-2,6 sialic acid linkages [33]. This diversity in linkage preferences suggests that, when present together, the three *C. perfringens* sialidases work in combination to generate free sialic acid, even from complex substrates [33]. 

## 3. *C. perfringens* Sialidases: Genetics and Regulation of Expression

All three *C. perfringens* sialidases are encoded by chromosomal genes, although those genes are located in different regions of the chromosome [38,39]. The sialidase-encoding ORFs in many *C. perfringens* strains have now been sequenced. Those sequencing analyses indicated that, amongst different *C. perfringens* strains, the NanJ sequence shares 96% to 100% identity, the NanI sequence has 98% to 100% identity, and the NanH sequence shares 93% to 100% identity [34,38,39]. 

As true for their toxin production, *C. perfringens* strains vary in their patterns of sialidase production. Most strains produce all three sialidases, with NanI usually being responsible for most of the sialidase activity in culture supernatants of those *C. perfringens* strains [34,36]. However, NanI production is not essential for *C. perfringens* growth since some strains of this bacterium naturally lack the *nanI* gene [36]. Interestingly, the *nanI* gene is consistently absent from the type A FP strains carrying a chromosomal *cpe* gene, as well as the genetically related type C darmbrand strains [36]. In contrast, the *nanI* gene is carried by most plasmid *cpe*-carrying type A AAD strains, *cpe*-negative type A normal human intestinal flora strains, and type C pig-bel strains [36]. Since NanI is usually the major sialidase of *C. perfringens*, it is not surprising that exosialidase activity is typically significantly lower for naturally *nanI*-negative strains compared to strains carrying a *nanI* gene [36]. The potential pathogenic importance of these differences will be discussed later. 

Most type A FP strains with a chromosomal *cpe* gene lack the *nanJ* gene, as well as the *nanI* gene. However, those typical FP strains do carry the *nanH* gene. It should be noted that occasional strains of *C. perfringens* besides the type A chromosomal *cpe* FP strains or type C darmbrand strains also lack a sialidase gene. For example, Strain 13 is a *cpe*-negative type A strain that can cause gas gangrene yet it lacks the *nanH* gene [37,40]. 

**Figure 2:**
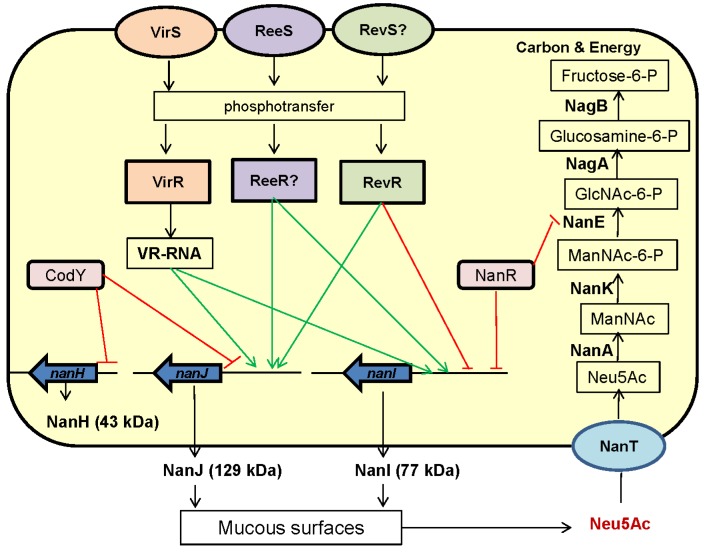
A proposed model for regulation of expression of sialidase genes and the pathway for sialic acid metabolism in *C. perfringens*. Exosialidases generate free sialic acid from mucus or host cell surfaces [33,40]. The free sialic acid is then transported into *C. perfringens* where it is metabolized to fructose-6-P [40]. There is evidence that VirS/VirR, RevR, ReeS, NanR, and CodY systems directly or indirectly affect sialidase production, although inter-relationships between these regulators are unclear [40,41,43,44,45,46]. The VirS/VirR two component system acts as a positive regulator of the *vrr* gene, which encodes VR-RNA. VR-RNA is then a positive regulator of *nanI* and *nanJ* expression [41,42]. The ReeS sensor kinase positively regulates *nanI* and *nanJ* gene expression, presumably by a putative transcriptional regulator named ReeR [43]. RevS positively regulates *nanJ* expression but negatively regulates *nanI* expression [44]. CodY represses *nanH* and *nanJ* expression [45]. Based on sequence homology comparisons with similar regulators in other bacteria, NanR may repress *nanI* expression and the sialic acid metabolism pathway, but there is no direct experimental evidence yet to support this hypothesis. Green lines indicate positive regulation while red lines indicate negative regulation.

The regulation of sialidase production by *C. perfringens* is complicated (Figure 2). A number of regulators influencing expression of one or more sialidase genes have been identified. For example, the VirS/VirR two-component signal transduction system was shown to upregulate *nanI* and *nanJ* expression [41]. Rather than a direct regulatory effect involving VirS binding to sialidase gene promoters, this VirS/R two-component system positively controls expression of the *vrr* gene, which encodes *virR*-regulated RNA (VR-RNA). This regulatory RNA then modulates sialidase gene expression [42]. ReeS is another sensor kinase whose presence also increases *nanI* and *nanJ* sialidase gene expression by a putative response regulator, referred to as ReeR [43]. In contrast, a transcriptional regulator named RevR has different regulatory effects on *nanJ* and *nanI* expression, possibly via a proposed sensor kinase named RevS. RevR increases *nanJ* expression, but negatively regulates *nanI* expression [44]. Finally, using a *codY* null mutant of *C. perfringens* type D strain CN3718, our group showed that CodY represses NanJ and NanH production, although it does not affect NanI production [45]. 

No proven regulator has yet been identified that directly modulates sialidase production by binding to the promoters of *C. perfringens* sialidase genes. However, gel mobility shift assays have demonstrated high affinity binding of a purified protein named NanR to DNA from the promoter region of the *nanI* gene [40]. Since NanR has homology with the ribose-5-phosphate isomerase B regulator (RpiR) family of transcriptional repressors known to control sialidase production in bacteria such as *E. coli, Vibrio vulnifcus*, and *Staphylococcus aureus*, this result suggests that NanR may be involved in *nanI* expression regulation [31,40,47]. Consistent with its potential involvement in sialic acid generation and usage, NanR lies within a six-gene operon encoding the complete pathway for transport and metabolism of sialic acid by *C. perfringens* [40]. As discussed in more detail below, this operon also encodes NanE (epimerase) and NanA (sialic acid lyase) enzymes. 

For the *nanI* and *nanJ* genes, primer extension analyses identified three or two putative transcription start sites, respectively [40]. These promoters are located within ~500 bp of the start codons of *nanI* and *nanJ*. This multiplicity of promoters may provide one explanation for why so many different regulators control sialidase expression, although (as mentioned) detailed understanding of regulator binding to these promoter sequences is currently lacking [40]. 

Only a limited number of bacterial species, mainly those having a close association with the sialic acid-rich environment of the host, can utilize Neu5Ac [48]. *C. perfringens* was actually the first bacterium demonstrated to be capable of utilizing a sialic acid (Neu5Ac) as a carbon source [31]. This result was later confirmed by another study showing that Neu5Ac can be used by *C. perfringens* when growing in a semi-defined medium [31]. It was also recently found that NanI and NanJ, but not NanH, can cause the release of sialic acid from Caco-2 cells [33]. This effect may contribute to pathogenesis, since these sialidases could help *C. perfringens* obtain nutrients in vivo by releasing sialic acid from glycolipids or glycoproteins on the host cell surface or in mucus. Interestingly, contact with Caco-2 cells was shown to upregulate the expression of NanI [34], which could potentiate NanI contributions during intestinal infections, as discussed later. 

NanI, but not NanJ, production is induced by the addition of Neu5Ac to a medium [40]. As introduced earlier, NanR is part of an operon that encodes a complete pathway for the transport and metabolism of sialic acid. After sialic acid is generated by sialidases, it is then transported and metabolized by products of this operon, also referred to as the Nan cluster. This process involves an initial conversion of sialic acid to N-acetyl glucosamine, followed by metabolism of that carbohydrate to fructose-6-P, which *C. perfringens* can use as carbon sources and for energy production (Figure 2) [40,46]. 

Many other pathogenic and commensal bacteria found in the intestines carry a Nan cluster for sialic acid utilization [48]. Examples of such bacteria include *Vibrio cholerae* and *Salmonella enterica* [48,49]. Many of these bacteria capable of utilizing sialic acid also colonize the human or animal intestinal tract. This correlation is probably not a coincidence since sialic acid is a component of mucin, the major protein in mucus, which is abundant in the human and animal intestines [48]. However, to generate free sialic acid from mucus for uptake and metabolism inside the bacterial cell, extracellular sialidases must be present in the intestinal environment. Interestingly, some pathogenic bacteria, e.g., *C. difficile*, have a Nan cluster but do not produce their own sialidase. Instead, it is believed that *C. difficile* uses free sialic acid generated in the intestines by sialidases produced by other bacteria, such as *Bacteroides thetaiotaomicron* [50,51]. 

## 4. Possible Contributions of Sialidases to *C. perfringens* Diseases 

Some pathogens use sialic acids to coat their cell surface, the flagellum, the capsule polysaccharide, or the lipopolysaccharide. This masks these bacteria so they can avoid the host immune system defense [28]. Whether *C. perfringens* coats its surface with host-derived sialic acid has not been studied, to our knowledge.

Sialidases can also promote in vivo growth and colonization of bacterial pathogens [30]. For extraintestinal infections, an example is the major human respiratory tract pathogen *Streptococcus pneumoniae,* which encodes up to three sialidases, named NanA, NanB, and NanC. NanA is the predominant sialidase that removes the sialic acid Neu5Ac from a variety of glycoconjugates. NanB is an intramolecular trans-sialidase producing 2,7-anhydro-Neu5Ac selectively from α2,3-sialosides, while NanC produces 2-deoxy-2,3-didehydro-*N*-acetylneuraminic acid (Neu5Ac2en), which can be hydrated to Neu5Ac. The three pneumococcal sialidases share a common catalytic mechanism up to the final product formation step, and all three sialidases are implicated in pathogenesis, including colonization, and are potential drug targets [52]. *S. pneumoniae* then takes up and metabolizes sialic acid using a similar pathway as present in *C. perfringens* [40].

The contribution of sialidases to growth and colonization during extraintestinal infections caused by *C. perfringens* is unclear. One study demonstrated that a *nanJ* and *nanI* double null mutant of *C. perfringens* strain 13 remains fully virulent in the mouse myonecrosis model [37]. This result could suggest that Neu5Ac metabolism is not essential for growth or colonization by *C. perfringens* in muscle. However, as noted in that study [37], this result does not necessarily preclude subtle contributions of sialidases to colonization or growth since the mouse myonecrosis model requires a massive inoculum that may mask such contributions. 

Increasing evidence indicates that sialidases and sialic acid often play significant roles in growth and colonization of the intestines by bacterial pathogens [34,49,52,53]. For example, sialic acid appears to be an important source of carbon and energy for survival, growth and adherence of *E. coli* in the gastrointestinal system [54]. Similarly, catabolism of sialic acid by *V. cholerae* plays a significant role in both in vitro and in vivo colonization and growth [49]. In addition, *V. cholerae* sialidase enhances the binding and uptake of cholera toxin [55]. In *V. vulnificus*, a Nan utilization system is also important for bacterial colonization and growth in the intestines [56]. The sialidase NanS play a role in *Clostridium sordellii* adhesion and enhances Non-TcsL mediated cytotoxicity [53]. As mentioned above, *C. difficile* uses sialic acid generated by sialidases of other bacteria to grow and persist in the intestines [50,51]. 

As mentioned earlier, NanI may contribute to in vivo growth. Contact of *C. perfringens* with Caco-2 cells increases the production of this enzyme [34] and NanI can induce the release of free sialic acids from enterocyte-like Caco-2 cells [33]. Since *C. perfringens* possesses a sialic acid utilization system, similar NanI-generated sialic acid in the intestines should be useful for in vivo growth of this bacterium.

Earlier studies demonstrated that NanI production also impacts the production of several *C. perfringens* toxins [34,37,57]. Specifically, inactivating the *nanI* gene in *C. perfringens* type A strain 13 caused a slight increase in supernatant activities of alpha toxin and perfringolysin O (PFO), which are contributors to gas gangrene [37], but their role (if any) in most intestinal infections, particularly human infections, is unsettled [3]. However, it was later demonstrated that inactivating the *nanI* gene in type D strain CN3718 reduced ETX toxin production in vitro and that either genetic or physical complementation could recover this ETX production [57]. NanI effects on ETX production involved reductions in both *codY* and *ccpA* transcript levels, suggesting a model whereby NanI generates sialic acid release in the intestines and that free sialic acid then alerts a type D strain of its presence in the intestines, making it worthwhile for this bacterium to upregulate ETX production to induce disease. 

There have been only limited studies to date on the effects of *C. perfringens* sialidase on toxin action, particularly on those toxins important for intestinal infections. An exception is ETX, where conclusions from early studies had been contradictory. One early study [58] reported that sialidases enhance ETX cytotoxicity towards Madin-Derby Canine Kidney (MDCK) epithelial cells, while a second study [59] reported that pretreating synaptosomal membranes with sialidases lowers their subsequent ETX binding levels. Recently a study used purified NanI, as well as *nanI, nanJ,* or *nanH* single null mutants, a *nanI/nanJ* double null mutant, and a triple sialidase null mutant, to study possible sialidase enhancement of ETX action [34]. Results obtained [34] conclusively showed that NanI sialidase increases the ETX sensitivity of MDCK cells and that this effect involves an increase in ETX binding levels. The mechanism of this enhancement is not yet clear but it could involve either NanI increasing the exposure of ETX receptors on the host cell surface or NanI modifying the host cell surface charge to increase binding of this toxin. Whether a similar effect extends to other *C. perfringens* toxins active during intestinal infections is not yet clear. 

Only limited studies have addressed the mechanism of *C. perfringens* adherence to host cells and tissues [60,61,62]. For example, the adhesins used by *C. perfringens* in vivo remain unclear. However, some strains produce a collagen adhesion protein (CNA), and/or fibronectin binding proteins (FbpA, FbpB) that have been implicated in adhesion and colonization [60,61,62,63]. 

**Figure 3:**
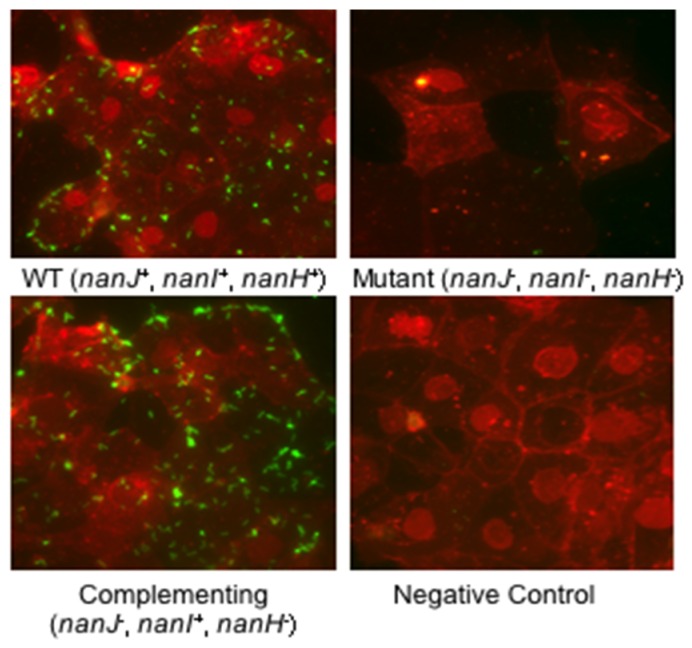
Adhesion of *C. perfringens* CN3718 to Caco-2 cells [34]. Caco-2 cells were incubated for 2 h at 37 °C under anaerobic conditions. *C. perfringens* CN3718 produces all three sialidases. This wild-type (WT) strain, and a complementing strain that produces only NanI, attach very well to Caco-2 cells as detected by immunofluorescence microscopy (600×). In comparison, only a few cells of an isogenic mutant with all three sialidase genes disrupted were able to attach to Caco-2 cells. Furthermore, when the triple mutant was complemented to produce only NanJ or NanH, those bacteria remained poorly adherent (not shown). Green: *C. perfringens*; Red: Caco-2 cells. Reproduced with permission from [34]. Creative Commons License 2011, Copyright J. Li and B.A. McClane.

Sialidases are known to contribute to the ability of some bacterial pathogens to adhere to host cells, tissues, or mucosal surfaces in the airways or intestines [30]. A recent in vitro study suggests sialidase contributions to *C. perfringens* intestinal adherence [34]. That study [34] demonstrated that type D strain CN3718 attaches to cultured Caco-2 enterocyte-like cells. This adhesion was specific since this intestinal disease strain exhibited significantly less adherence to fibroblasts and kidney cells [34]. Interestingly, the attachment of CN3718 to Caco-2 cells is greatly facilitated by NanI production (Figure 3). Wild-type CN3718 exhibited much more adherence to Caco-2 cells compared to an isogenic triple mutant strain that does not produce any sialidase. Complementation studies showed that, of the three sialidases made by CN3718, restoring NanI production to the triple sialidase mutant yielded the greatest enhancement of adherence. That result indicated that NanI is of prime importance for sialidase enhancement of CN3718 attachment to Caco-2 cells [34]. 

However, NanI itself does not appear to be a significant adhesin since the CN3718 *codY* null mutant shows 50% less adherence to Caco-2 cells compared to wild-type CN3718, despite this mutant producing the same levels of NanI as the wild-type strain [45]. This result suggests a two-step adherence mechanism. First, secreted NanI modifies the Caco-2 enterocyte-cell surface, which then allows the unknown adhesin present on the surface of *C. perfringens* to more easily bind to the still unidentified receptor(s) on the enterocyte-cell surface. 

Sialidases may enhance *C. perfringens* adherence to host cells via both nonspecific and specific mechanisms. Sialic acids, typically present at the distal ends of carbohydrate chains, are negatively charged carbohydrates [28,30]. In addition, terminal sialic acids promote endothelial barrier integrity, so treatment of epithelial monolayers with *C. perfringens* sialidases may lead to barrier disruption and may increase access for *C. perfringens* so they can adhere [64]. Therefore, nonspecific effects of secreted NanI on both charge and epithelial barrier integrity could help to increase toxin binding and *C. perfringens* colonization. A *C. perfringens* sialidase (not specified) has, in fact, been shown to affect barrier resistance in some, but not all, host cells [64]. However, for some cell lines, nonspecific effects do not appear to completely explain NanI enhancement of *C. perfringens* adherence. As mentioned, *C. perfringens* CN3718 exhibits much greater adhesion for certain mammalian cells, such as Caco-2 and HT-29 intestinal cell lines, than for cell lines of nonintestinal origin [34]. Similarly, the toxins involved in *C. perfringens* intestinal infections only bind to and affect certain cells. Those observations suggest that NanI can also have specific effects in promoting *C. perfringens* adherence and toxin binding. This could involve specific effects of NanI sialidase on modifying host cell surface adhesins/toxin receptors and/or trimming back nearby molecules to better unmask the adhesin or toxin receptor on host cell surfaces. 

When *C. perfringens* causes diseases originating in the intestines, its secreted proteins come into contact with host proteases, such as trypsin, that are present in the intestinal lumen. While some *C. perfringens* proteins, e.g., CPB, are highly sensitive to intestinal proteases [16], other proteins secreted by this bacterium are proteolytically-activated in the intestines. A prime example of a proteolytically-activated *C. perfringens* protein is ETX, which is initially produced as an inactive prototoxin and then activated by intestinal trypsin, chymotrypsin, and carboxypeptidases that remove N- and C-terminus residues from the prototoxin [65]. Similarly, trypsin or chymotrypsin treatment of CPE also increases cytotoxicity, in this case by removing N-terminal sequences from the native CPE protein to facilitate toxin oligomerization during pore formation [66]. Interestingly, NanI (but not NanJ or NanH) can also be proteolytically-activated by trypsin [33,34]. The increased enzymatic activity of trypsin-activated NanI was shown to be substrate-specific [33]. Collectively, these observations suggest that trypsin activation of NanI may contribute to *C. perfringens* intestinal diseases. 

**Figure 4:**
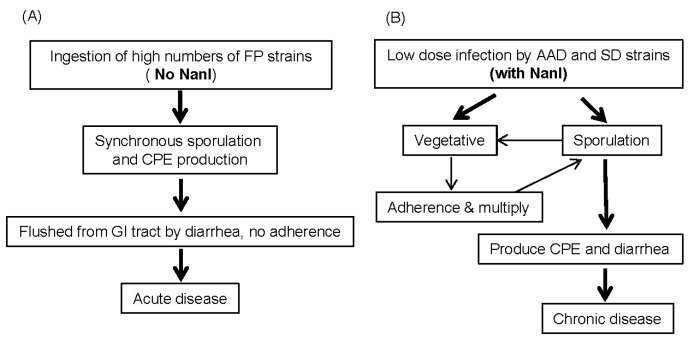
Possible models for acute *C. perfringens* type A FP vs. chronic gastrointestinal diseases like AAD. (**A**) *C. perfringens* type A FP does not require NanI production because strains are ingested in large amounts, sporulate in vivo to produce CPE and are then quickly removed from the intestines by diarrhea. (**B**) *C. perfringens* type A AAD does involve NanI production, which may promote adherence and colonization, as required for chronic diarrhea. Modified with permission from [36]. Copyright 2014 American Society for Microbiology.

While NanI can enhance *C. perfringens* adherence and toxin binding, typical type A FP strains, i.e., those with a chromosomal *cpe* gene, do not carry the *nanI* gene. Furthermore, those typical FP strains possess very low exosialidase activities [36]. The limited production of sialidases by type A FP strains obviously does not hinder their ability to cause food-borne intestinal diseases. Unlike the typical FP strains, nearly all type A AAD strains do carry a *nanI* gene. When a *nanI* null mutant of a type A AAD strain was characterized, this *nanI* null mutant was shown to enter the sporulation cycle earlier and to produce more CPE than its wild-type parent. This finding supports the dispensability of NanI for the typical FP strains, which cause an acute disease. During the acute FP, NanI would not be needed for nutritional purposes or to enhance bacteria adherence or colonization since the typical FP strains are ingested in very large numbers in contaminated food, then quickly sporulate in the intestines to produce CPE, induce diarrhea via that CPE, and exit from the intestines via the flushing action of diarrhea (Figure 4) [36]. In contrast, type A AAD or sporadic diarrhea (SD) strains with a plasmid *cpe* gene cause chronic gastrointestinal disease lasting up to several weeks, so in vivo growth and colonization is important for their persistence. Those Type A AAD or SD strains may use NanI for their growth and colonization, as necessary to achieve the persistence required to cause a chronic diarrhea (Figure 4) [36].

## 5. Sialidase Inhibitors: Potential Therapeutic Agents?

As described above, sialidases may offer several contributions to *C. perfringens* pathogenesis, particularly during intestinal infections. *C. perfringens* diseases are challenging to treat with antibiotics because already-synthesized toxins will continue to work even after the administration of antibiotics. Similarly, it is often difficult to treat or prevent *C. perfringens* infections with vaccines or neutralizing antibodies because some individual strains produce multiple (up to five) different toxins [2,3]. Those factors suggest that sialidase inhibitors represent interesting potential candidates for drug development. 

Sialidase inhibitors have proven to be efficacious for the treatment of some infectious disease [67]. The most notable clinical application of sialidase inhibitors are several sialidase inhibitors (Zanamivir, Oseltamivir, Peramivir (Rapivab)) that are currently used for treating infections involving influenza virus [67]. However, there is also precedent for sialidase inhibitors interfering with bacterial growth and adhesion. Oseltamivir reduced the growth and adherence of *Tannerella forsythia*, which causes periodontitis, by two- to three-fold [68]. 

Two classic sialidase inhibitors, i.e., Siastatin B (SB) and N-acetyl-2,3-dehydro-2-deoxyneuraminic acid (NADNA), have been tested against *C. perfringens* [33,36,57,69,70]. SB, a broad-spectrum sialidase inhibitor isolated from a *Streptomyces* spp. culture, is an unusual 6-acetamido-3-piperidinecarboxylate [70]. NADNA is an analogue of 2-deoxy-2,3-didehydro-N-acetylneuraminic acid that is modified at the C-4 position. NADNA can inhibit the sialidase activity from influenza viruses A and B, parainfluenza 2 virus, *Vibrio cholerae*, *Arthrobacter ureafaciens*, *C. perfringens*, and sheep liver [69]. 

A recent study measured the effects of SB and NADNA on sialidase activities of overnight culture supernatants of *C. perfringens* CN3718 mutants ENanJ, ENanI, or ENanH, which contain natural levels of only NanJ, NanI, or NanH, respectively [33]. NanI was the most sensitive sialidase to both NADNA and SB, while NanH was the least sensitive sialidase to these inhibitors; IC_50_s for these inhibitors are shown in Table 3 [33]. 

It was also shown that the adherence of *C. perfringens* AAD strain F4969 to Caco-2 cells can be reduced by either SB or NADNA [36]. In addition, both NADNA and SB efficiently inhibited sialidase activity in bacterial cell-free supernatants collected from *C. perfringens* type D strain CN3718. Consistent with those results, CN3718 cultures grown in the presence of the SB inhibitor also exhibited substantially reduced culture sialidase activity and ETX production. However, the NADNA sialidase inhibitor did not inhibit sialidase activity in CN3718 cultures, even at very high doses of the inhibitor. In the presence of NADNA, CN3718 cultures still possessed strong sialidase activity and also made the same amounts of ETX as wild-type CN3718 grown in the absence of any sialidase inhibitor [57]. Collectively, these results suggest that at least some sialidase inhibitors may be potentially useful therapeutics against *C. perfringens* infections.

## 6. Concluding Remarks and Future Directions

Most studies of *C. perfringens* virulence have, correctly, focused on toxin contributions. Only a few in vivo studies have thus far examined the role of exoenzymes in *C. perfringens* pathogenesis and their results have been equivocal. However, those studies examined the possible role of sialidases in gas gangrene. Recent in vitro studies suggest that sialidases, particularly NanI, may be important contributors to *C. perfringens* intestinal infections. Those contributions could include upregulated production of some toxins relevant to intestinal infections, enhanced binding and activity of some of those toxins, increased adherence of *C. perfringens* to host cells, and possibly generation of substrates for growth and metabolism [33,34,36,57]. There is a clear need to move these promising findings into appropriate animal models to firmly evaluate sialidase contributions to *C. perfringens* virulence. 

In vitro studies also suggest that some sialidase inhibitors might be useful therapeutics for treating *C. perfringens* infections originating in the intestines [57]. This possibility also needs to be evaluated in animals. Identification of more potent inhibitors of *C. perfringens* sialidases may be helpful for those studies. 

## Figures and Tables

**Table 1:** *C. perfringens* typing table.

Type	Toxin Production ^a^			
	α	β	ε	ι
A	+	−	−	−
B	+	+	+	−
C	+	+	−	−
D	+	−	+	−
E	+	−	−	+

^a^ + indicates production of that toxin, while – indicates no production of that toxin.

**Table 2:** Diseases associated with the major types/subtypes of *C. perfringens.*

Toxinotypes ^a^	Subtype	Most Significant Diseases ^b^
A	No CPE ^c^ or NetB production	Human and animal myonecrosis (gas gangrene)
	NetB-producing	Necrotic enteritis of poultry
	CPE-producing	Human food poisoning and non-foodborne gastrointestinal disease
B		Necro-hemorrhagic enteritis of sheep (lamb dysentery)
C		Human enteritis necroticans (Darmbrand, pigbel); necrotic enteritis of neonatal individuals of several animal species (e.g., cattle, sheep, pigs)
D		Enterotoxemia of sheep and goats
E		Suspected association with gastrointestinal disease of cattle, sheep and rabbits

^a^ All types of *C. perfringens* may also produce several other toxins, including, but not limited to, beta2 toxin (CPB2), perfringolysin O (PFO), and toxin *C. perfringens* large cytotoxin (TpeL); ^b^ Only diseases that have been confirmed to be associated with each type of *C. perfringens* and significant in terms of prevalence are included in this table; ^c^ CPE is *C. perfringens* enterotoxin.

**Table 3:** Effect of sialidase inhibition on sialidase activity (IC_50_).

Sample	NADNA (IC_50_)	Siastatin B (IC_50_)
CN3718	18.9 µM	42.2 µM
ENanJ	12.4 µM	15.1 µM
ENanI	13.4 µM	27.5 µM
ENanH	44.6 µM	50.9 µM

Reproduced with permission from [33]. Copyright 2014 American Society for Microbiology.
